# Supplementary material for: Primary Ciliary Dyskinesia Patient-Specific hiPSC-Derived Airway Epithelium in Air-Liquid Interface Culture Recapitulates Disease Specific Phenotypes In Vitro
Source: Cells. 2023 May 24;12(11):1467. doi: 10.3390/cells12111467 (PMC10252476; doi:10.3390/cells12111467)
Supplement: Supplementary file 1 [file cells-12-01467-s001.zip › cells-2389792-supplementary.pdf]

Supplemental Material

Table S1. Duration of anterior foregut induction for different hiPS cell lines.

Cell line	Duration of AFE 1	Duration of AFE 2
WT	24h	24h
DNAH5^{mut} Cl22	48h	48h
DNAH5^{mut} Cl24	48h	48h
NME5^{mut}	48h	48h

Table S2. Assay IDs and primer sequences for qRT-PCR.

Target Gene	Assay ID / Primer sequence
CCDC40	qHsaCED0045023
CK5	qHsaCED0005398
FOXJ1	FW: AGCCAGGCACCACATACTTA<br>REV: GCTCTCCGCCATGTCTGC
P63	FW: TCATTTGATTCGAGTAGAGGGGAAC<br>REV: CTCCAACACAACTGCTGTTACACAT
NKX2.1	FW: CGGCATGAACATGAGCGGCAT<br>REV: GCCGACAGGTACTTCTGTTGCTTG
NGFR	FW: GCCAGGACAAGCAGAACAC<br>REV: CGTGTAATCCAACGGCCAG
MUC5AC	FW: GCACCAACGACAGGAAGGATGAG<br>REV: CACGTTCCAGAGCCGGACAT
CCSP	FW: GGACACCCTCCCCCAAAAGC<br>REV: TGGTGGGCGTGGACTCAAAG
betaActin	FW: ATTGCCGACAGGATGCAGAA<br>REV: GGGCCGGACTCGTCATACTC
GAPDH	FW: CCATCTTCCAGGAGCGAGATC<br>REV: GCAGAGATGATGACCCTTTTGG

Table S3. primary antibodies.

Antibody	Vendor, RRID, Dilution
anti-MUC5AC	Thermo Fisher Scientific, RRID: AB_10978001; 1:200
anti-acetylated-tubulin (AcTub)	Cell Signaling Technology, RRID: AB_10544694; 1:800
anti-CCSP (CC10)	Santa Cruz Biotechnology, RRID: AB_10915481; 1:200
anti-cytokeratin 5 (CK5)	Abcam, RRID: AB_869890; 1:300
anti-p63	Abcam, RRID: AB_305870; 1:300
anti-AcTub	Sigma-Aldrich, RRID: AB_609894; 1:2000
anti-DNAH5	Atlas Antibodies, RRID: AB_10672348; 1:500
anti-NME5	Sigma-Aldrich, RRID: AB_10960286; 1:400
anti-RSPH1	Sigma-Aldrich, RRID:AB_1858392; 1:200
anti-RSPH4A	Sigma-Aldrich, RRID:AB_10601612; 1:200
anti-RSPH11	Sigma-Aldrich, RRID:AB_10673415; 1:200
anti-CXCR4	Thermo Fisher Scientific, RRID: AB_1724113; 1:33.3
anti-cKIT	Thermo Fisher Scientific, RRID:AB_2043797; 1:33.3

anti-EpCAM	BD Biosciences, RRID: AB_400262; 1:25
anti-NKX2.1	Miltenyi Biotec, RRID: AB_2751514; 1:2000

Table S4. secondary antibodies.

**Antibody**	**Vendor, RRID, Dilution**
donkey anti-mouse AF488	Jackson ImmunoResearch Labs, RRID:AB_2340846; 1:300
donkey anti-rabbit Cy5	Jackson ImmunoResearch Labs, RRID:AB_2340607; 1:300
donkey anti-mouse Cy5	Jackson ImmunoResearch Labs, RRID:AB_2340819; 1:300
donkey anti-rabbit AF488	Jackson ImmunoResearch Labs, RRID:AB_2313584; 1:300

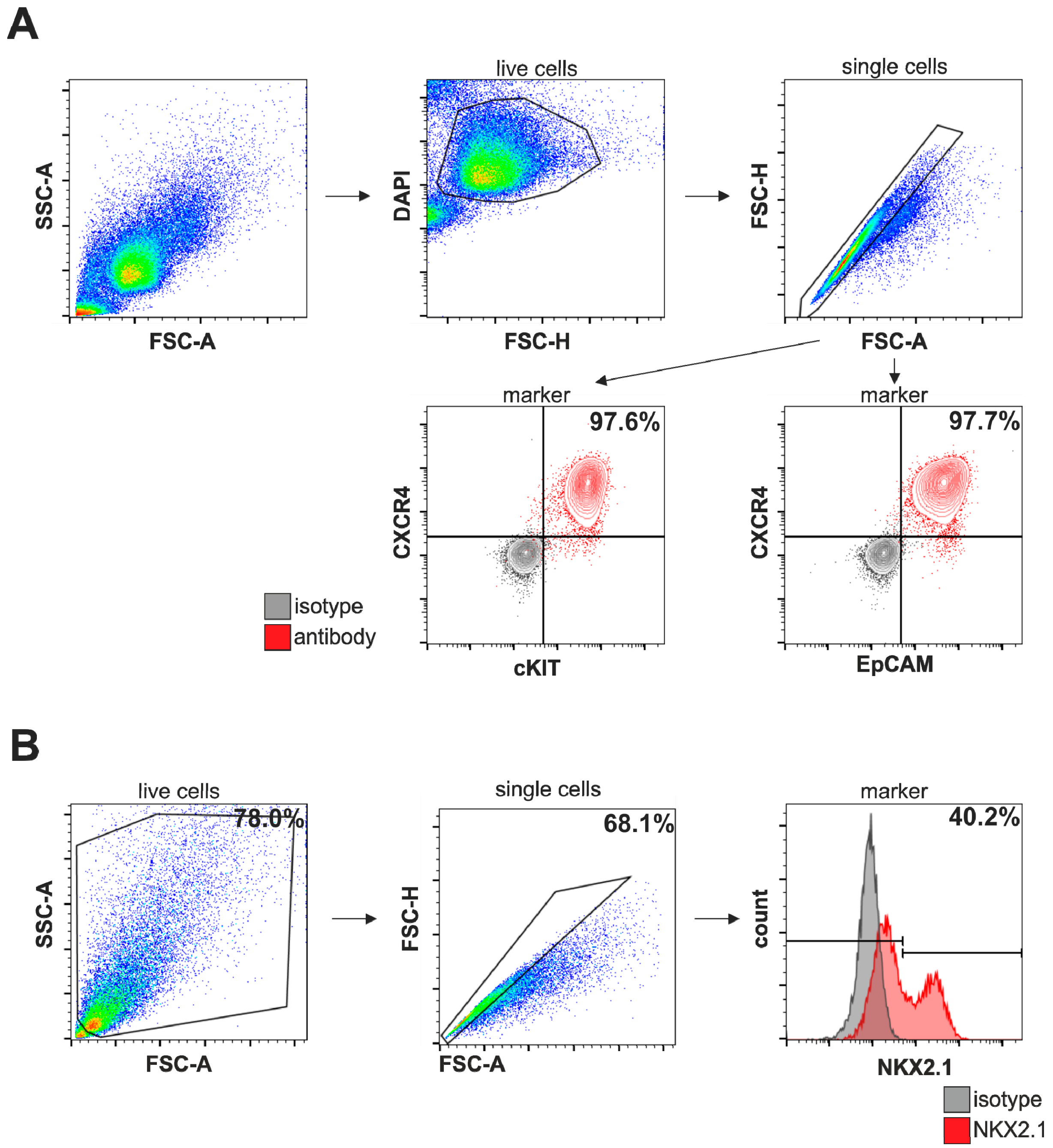

Supplemental Figure S1. Gating strategy for flow cytometric analysis. Gating strategy for identification of definitive endoderm (DE) cell population at day 3 of differentiation using antibodies against CXCR4, cKIT and EpCAM (red) and respective isotype controls (grey) (**A**). Gating strategy for identification of lung progenitor cells at day 14 of differentiation using a NKX2.1-specific antibody (red) and respective isotype control (grey) (**B**).
