# Supplementary material for: Primary Ciliary Dyskinesia Patient-Specific hiPSC-Derived Airway Epithelium in Air-Liquid Interface Culture Recapitulates Disease Specific Phenotypes In Vitro
Source: Cells. 2023 May 24;12(11):1467. doi: 10.3390/cells12111467 (PMC10252476; doi:10.3390/cells12111467)
Supplement: Supplementary file 1 [file cells-12-01467-s001.zip › cells-2389792-supplementary.pptx]

## Slide 1
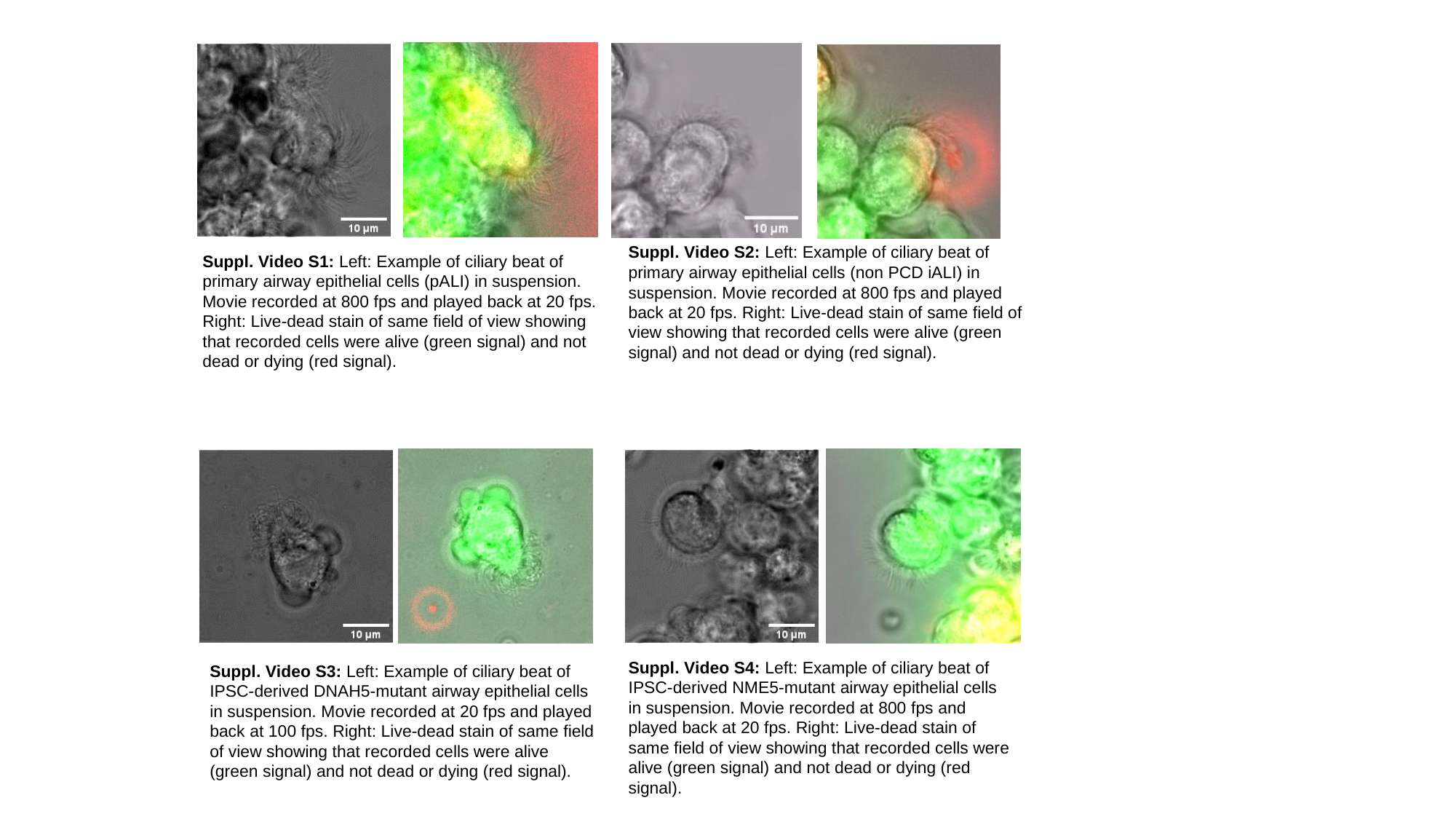

Suppl. Video S2: Left: Example of ciliary beat of primary airway epithelial cells (non PCD iALI) in suspension. Movie recorded at 800 fps and played back at 20 fps. Right: Live-dead stain of same field of view showing that recorded cells were alive (green signal) and not dead or dying (red signal).
Suppl. Video S1: Left: Example of ciliary beat of primary airway epithelial cells (pALI) in suspension. Movie recorded at 800 fps and played back at 20 fps. Right: Live-dead stain of same field of view showing that recorded cells were alive (green signal) and not dead or dying (red signal).
Suppl. Video S4: Left: Example of ciliary beat of IPSC-derived NME5-mutant airway epithelial cells in suspension. Movie recorded at 800 fps and played back at 20 fps. Right: Live-dead stain of same field of view showing that recorded cells were alive (green signal) and not dead or dying (red signal).
Suppl. Video S3: Left: Example of ciliary beat of IPSC-derived DNAH5-mutant airway epithelial cells in suspension. Movie recorded at 20 fps and played back at 100 fps. Right: Live-dead stain of same field of view showing that recorded cells were alive (green signal) and not dead or dying (red signal).
